# Microencapsulation of nattokinase from fermentation by spray drying: Optimization, comprehensive score, and stability

**DOI:** 10.1002/fsn3.2378

**Published:** 2021-06-08

**Authors:** Ganlu Li, Tao Li, Feng He, Cheng Chen, Xu Xu, Weilong Tian, Yue Yang, Xun He, Hui Li, Kequan Chen, Ning Hao, Pingkai Ouyang

**Affiliations:** ^1^ College of Biotechnology and Pharmaceutical Engineering Nanjing Tech University Nanjing China; ^2^ Jiangsu Jicui Industrial Biotechnology Research Institute Co. Ltd, Nanjing China

**Keywords:** microencapsulation, nattokinase, response surface methodology, spray drying, storage stability

## Abstract

Nattokinase from fermentation has recently gained more attention due to its beneficial effects on cardiovascular system. However, the instability of free nattokinase limits its application. The aim of the study was to develop a spray‐drying microencapsulation process to obtain the nattokinase powder with high activity, high quality, and strong storage stability. Hence, the microencapsulation process of nattokinase from fermentation by spray drying was optimized. Experiments of single‐factor and response surface methodology were used to assess the comprehensive scores and nattokinase activities. According to single‐factor and response surface methodology results, optimum parameters of microencapsulation process of the nattokinase power by spray drying were 30% of mass ratio of wall materials, 139°C of air inlet temperature, 8 L/h of feed rate, and 80°C of outlet temperature. The final optimized result encompassed a comprehensive score of 96, nattokinase activity of 1,340 IU/ml, and moisture content of 4.1 ± 0.1%. In addition, the microencapsulated nattokinase power showed strong storage stability in the conditions of different temperatures and pH. After 30 days of storage, the nattokinase powder was still white or light yellow, with a special smell, no peculiar smell and paste taste, and no impurity. These results build the basis of further industrialization of the nattokinase powder from fermentation broth by spray drying.

## Graphical abstract

A spray‐drying microencapsulation process to obtain the nattokinase powder with high activity, high quality and storage stability.

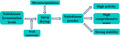

## 1 INTRODUCTION

Nattokinase, an alkaline serine protease of the subtilisin family, possesses various crucial beneficial effects on cardiovascular system for improving thrombolysis because of its preventive and therapeutic effects, safety, small side effects, and low price (Amin et al., 2020; Ni et al., 2016). Importantly, nattokinase is easy to be absorbed efficiently by the gastrointestinal tract with the strong thrombolytic activity and fibrinolytic activity induced after its oral administration (Liu et al., 2019; Weng et al., 2017). A single dose of nattokinase administration showed the enhanced fibrinolysis and anticoagulation profiles (Kurosawa et al., 2015). Nattokinase is mainly produced by the *Bacillus* *subtilin* (natto) during the fermentation of soybeans or soybeans‐based ingredients to produce Natto‐containing foods (Cai et al., 2017; Man et al., 2019). Now, Nattokinase is typically used as a functional factor in health functional food to prevent cardiovascular diseases and thrombosis. However, free nattokinase is sensitive to high‐temperature and alkaline and acidic environment that reduce its storage stability and the medical effect after oral administration (Devi et al., 2016). Due to the easy inactivation denaturation of free nattokinase, folic acid‐modified chitosan modified nattokinase showed the enhanced stability and fibrinolysis activity (Chen et al., 2014). However, its operation process was complex and expensive. Therefore, improving the stability of nattokinase in a simple and economic way can significantly enhance its application value.

Spray drying has become a crucial technique that is widely used to ameliorate the stability of food ingredients, like proteins, enzymes, colorants, oil, antioxidants, and cells, by transforming the liquid state into a solid powder (Alcântara et al., 2019; Bustamante et al., 2020; Obón et al., 2020; Rezvankhah et al., 2020; Wang & Selomulya, 2020; Yingngam et al., 2019). Spray drying was used to produce the blueberry‐polyphenol food ingredients, which found that the complexed polyphenols structural integrity and biological activities are retained (Hoskin et al., 2019). The spray drying of curcumin encapsulated in soy protein isolate microencapsulation showed the enhanced bioaccessibility and bioactivities (Chen et al., 2020). Because of high drying temperatures and atomization process causing protein instability, process parameters, like as feed rate, outlet temperature, and solid concentration of feed, are significant factors affecting the enzymatic activity. Understanding the critical process and formulation parameters are required for the successful spray drying of biomolecules (Ziaee et al., 2020). In process operability of fabrication of the uniform enzyme‐immobilized carbohydrate microparticles, spray drying offered a rather simple and economical means to produce enzymatic microparticles of high activity with the appropriate dosage of carbohydrate. The enzyme‐immobilized carbohydrate microparticles formed the hydrogen bonding between enzyme and carbohydrate and were highly amorphous glass matrices, which decrease enzyme unfolding and aggregation (Zhang et al., 2018). Encapsulation of ascorbyl palmitate in maize starch by spray drying improved its storage stability under ultraviolet light conditions (Bamidele & Emmambux, 2019). Compared to freeze‐drying, spray drying is a convenient, cost‐effective, higher applicable, and scalable encapsulation procedure in the food and pharmaceutical industries (Rezvankhah et al., 2020). Spray drying is an interesting alternative means of increasing the stability of powdered foods and food ingredients (Bilušić et al., 2020; González et al., 2019). Freeze drying has been used to dry the nattokinase (Garg & Thorat, 2014). However, spray drying used for microencapsulating nattokinase is rarely reported.

In this work, the nattokinase produced by liquid fermentation of *B*. *subtilin* was used as raw material to study the effects of different processing parameters on the nattokinase activity and product quality of nattokinase power in spray drying for microencapsulating nattokinase. The spray drying process was optimized by a single‐factor and response surface analysis. It was found that the storage stability of nattokinase power is improved in high temperature and alkaline and acidic environment. This work provides reference for the application of heat‐sensitive enzyme powder in food.

## 2 MATERIALS AND METHODS

### 2.1 Materials

Nattokinase fermentation broth was made by our laboratory. Food‐grade modified starch was purchased from Juntao. Food‐grade β‐dextrin was got from Tianzhao. Urokinase (1,280 IU), thrombin (190 bp), and fibrinogen were purchased from Yuanye.

### 2.2 Preparation of nattokinase powder by microencapsulation

Wall materials were made in the food‐grade modified starch and food‐grade β‐dextrin mixed evenly according to the mass ratio of 2:1 (weight: weight) in reserve. Then, the wall materials were added slowly into the nattokinase fermentation broth. After that, it kept stirring for 30 min at 35–40°C. Finally, fermentation broth containing the wall materials was screened with 80 mesh sieves. Finally, a spray dryer (G15 model, 15 kg/h, cocurrent, Yuanyu) was used to prepare the nattokinase powder.

### 2.3 Determination of moisture content in nattokinase powder

A certain amount of nattokinase powder was weighed (*m*_1_), and dried to constant weight (*m*_2_) in the drying oven (GZX‐9140MBE, Boxun) at 100–105°C. The moisture content of nattokinase powder was calculated with Equation (1).

(1) $$\text{Moisture content}\;\%=\left(m_{1}‐m_{2}\right)/m_{2}\times 100$$

### 2.4 Determination of nattokinase activity

The activity of nattokinase was determined by the fibrin plate method. 7.5 ml of 2% wt agarose solution (pH 7.5) was taken and heated in 50°C at water bath (SHJ‐1, Deke) for 5 min, 225 ml thrombin solution (1 bp/ml, pH 7.5) was added, and mixed well and heated in 50°C at water bath for 5 min, then, 7.5 ml 3% wt fibrinogen solution (pH 7.5) was poured into heat preservation thrombin‐agarose solution and quickly mixed, poured into clean culture dish at room temperature for 1 hr, and then punched holes after solidification.

Standard curve preparation: urokinase was prepared into 2,000 IU/ml solutions with normal saline, and then diluted with normal saline to prepare 1,600, 1,400, 1,200, 1,000, 800, 600, 400, and 200 IU/ml solution. 10 μl samples were taken and put on the fibrin plate. After holding at 37°C for 18 hr, the diameter of dissolution circle on the fibrin plate was determined, and the standard curve was obtained (*Y* = 2.6506*X*‐74.001, *R^2^* = 0.9901).

Spray‐dried nattokinase powder samples (10 g) or equal mass free nattokinase were taken for dissolving into 200 ml normal saline and stirred 30 min, then centrifuged for 5 min at 12,000 rpm, then 10 μl samples were added to the fibrin plate, and incubated 18 hr at 37°C, the diameters of dissolution circle on the fibrin plate were measured, and the nattokinase activities were calculated according to the standard curve.

### 2.5 Comprehensive score

Comprehensive scores of the nattokinase powder samples were evaluated according to the method of Ma`s (2020). Briefly, twenty semitrained peoples were chosen to set up an evaluation group, and the color, smell, impurities, nattokinase activity, and moisture content were determined as the five sensory evaluation indexes of the spray‐dried nattokinase powder. The comprehensive scores of nattokinase powder were obtained by adding up the scores of 5 indexes of each experimental result. The specific evaluation criteria and scores are shown in Table 1.

**TABLE 1:** The standard for calculating the comprehensive score of nattokinase powder

Character	Standard	Score
Color	White or light yellow	16–20
	Burnt yellow or brown	10–15
	Blackened	0–10
Smell	Special smell, no peculiar smell	16–20
	Slight peculiar smell	10–15
	Peculiar smell	0–10
Impurities	No impurities	16–20
	Small amount of impurities	10–15
	More impurities	0–10
Nattokinase activity	>1,000 IU/ml	16–20
	800 ~ 1,000 IU/ml	10–15
	<800 IU/ml	0–10
Moisture content	<5%	16–20
	5 ~ 10%	10–15
	>10%	0–10

### 2.6 Single‐factor experiments

In this work, a series of single‐factor experiments were performed to study the influence of four factors, namely the mass ratio of wall material, inlet air temperature, feed rate, and outlet temperature, on the spray drying of nattokinase powder. To optimize every single factor, a single variable was determined in the following given scope. The mass ratios of wall material were tested at 20, 25, 30, 35, and 40% wt; the inlet air temperatures at 120, 130, 140, 150, and 160°C; the feed flow rates at 4, 6, 8, 10, and 12 L/h; the outlet temperatures at 60, 70, 80, 90, and 100°C, respectively. Then, the optimization variables were applied for the following evaluation of the nattokinase activities and comprehensive scores of the nattokinase powder.

### 2.7 Response surface methodology

By integrating the results of the single‐factor experiments, a response surface methodology (RSM) experiment was conducted based on a Box‐Behnken center combination design (CCD) with the mass ratio of wall material (*A*), inlet air temperature (*B*), feed rate (*C*), and outlet temperature (*D*) as experimental factors. With the comprehensive scores (*Y_1_*) and nattokinase activities (*Y_2_*) of the nattokinase powder as the response values, a 4‐factor and 3‐level CCD analysis were conducted to optimize the spray drying process parameters of the nattokinase powder. The experimental factors and levels are listed in Table 2.

**TABLE 2:** Designs and response of Box‐Behnken CCD experimental design

Level	*A*/%	*B*/°C	*C*/(L/h)	*D*/°C
−1	25	130	6	70
0	30	140	8	80
1	35	150	10	90

### 2.8 Storage stability

The nattokinase powder samples by microencapsulation were stored in the uncapped glass dish and placed in a dry thermostat (GD‐2010, Haixiang, Shanghai, China) at 25, 30, 40, and 50°C for storage stability test. The relative humidity was controlled at 20.0 ± 0.5%. The nattokinase activities of nattokinase powder samples were analyzed every 5 days.

The pH of nattokinase fermentation broths was adjusted to 5, 6, 7, 8, 9, and 10. After adding the wall material, nattokinase powders with different pH according to the optimum spray drying conditions were sprayed. Then, the nattokinase powder samples by microencapsulation with different pH were stored in the uncapped glass dish and placed in a dry thermostat at 25°C for storage stability test. The relative humidity was controlled at 20.0 ± 0.5%, and nattokinase activities of nattokinase powder samples were analyzed every 5 days.

### 2.9 Statistical analysis

All results were presented as mean ± standard deviation (*SD*). The data analysis was carried out by the student's *t* test with SPSS 20.0 and values with asterisks were statistically significant (*p* < .05).

## 3 RESULTS AND DISCUSSION

### 3.1 Effect of mass ratio of wall material on spray drying of nattokinase powder

**FIGURE 1:**
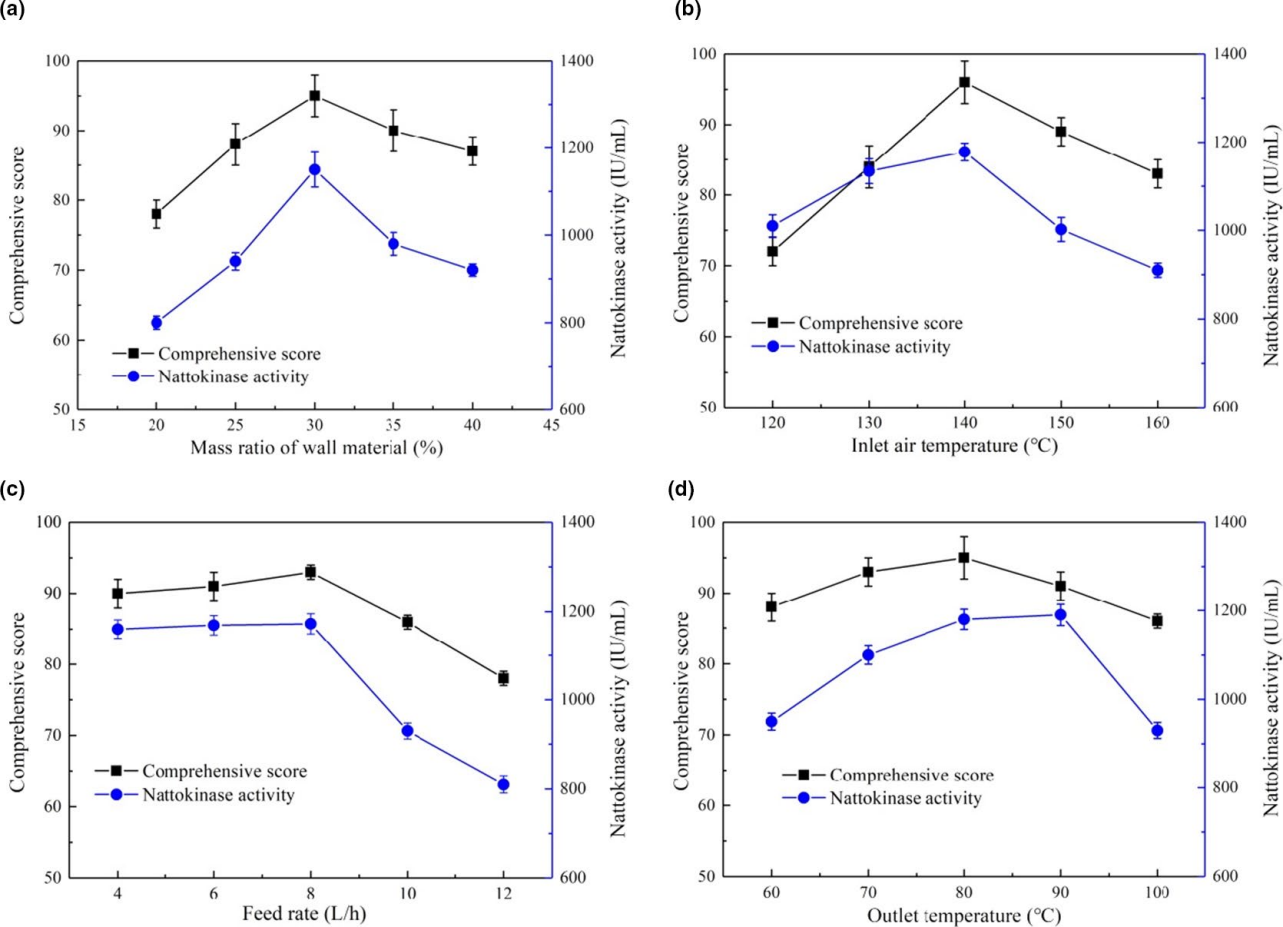
Effect of (a) mass ratio of wall material, (b) inlet air temperature, (c) feed rate and (d) outlet temperature on spray drying of nattokinase powder by microencapsulation

The sizes of the fog droplet and the diameters of powder are mainly adjusted by atomization process. Rotational speed of the atomizer is key parameter to control the atomization process (Chávez‐Salazar et al., 2019; Huang & Mujumdar, 2008). According to the pre‐experiment results, the rotational speed of the atomizer of G15 spray dryer was set at 20,000 rpm. Due to different drying wall materials with unique processing effects, we chose the food‐grade modified starch and the food‐grade β‐dextrin as two‐component wall materials to reduce the phenomenon of wall sticking. According to the results of pre‐experiment, the mass ratio of modified starch and β‐dextrin was set to 2:1 (weight: weight). The inlet air temperature was set at 140°C, the feed flow rate at 10 L/h, and the outlet temperature at 90°C. Spray drying of the nattokinase powder by microencapsulation was carried out under the conditions of mass ratios of wall materials of 20, 25, 30, 35, and 40%, respectively (Figure 1a).

With a small amount of wall materials, spray drying effect was poor and adhesion was particularly serious. In addition, if the mass ratio of wall materials was too much, it is easy to block the nozzle of the atomizer, and the nonuniform liquid droplets would cause the powder to have higher moisture content, easy to agglomerate, and reduce the quality of powder. The low mass ratio of wall materials would result in poor encapsulating effect on the enzyme, and easy to make the liquid droplets appear in the spray drying process and the local temperature too high for reducing the activity of the enzyme and the flavor (Bajaj et al., 2021; Ma et al., 2020). The powder yield was 97.5 ± 1.5%; however, adding too much or too little wall materials would lead to reduction targets product quality. Thus, it was necessary in order to identify the optimum amount of wall materials required in the spray drying process.

When the added mass ratios of wall materials were increased from 20%–30%, nattokinase activities (*p* < .05) and comprehensive scores (*p* < .05) of the nattokinase powder increased significantly (Figure 1a). However, excessive addition of wall materials in the range of 30%–40% reduced the nattokinase activities and comprehensive scores of the nattokinase powder. Moreover, it caused insufficient drying and increased the moisture content of the nattokinase powder. Low mass ratio of wall materials was prone to sticky walls during the spray drying process for the high viscosity of the material liquid, and the powder had a peculiar and paste smell of burnt‐brown phenomenon. However, with the increase in the mass ratios of wall materials, the moisture content of the nattokinase powder by microencapsulation was too high, which reduced the activity of nattokinase powder. The moisture content of the nattokinase powder less than 5.0% is in line with the quality requirements. Modified starch can accelerate more moisture loss during spray drying because of less number of exposed hydroxyl groups than unmodified starch (Ding et al., 2020). When the mass ratio of wall materials was 30%, nattokinase activity and comprehensive score of the nattokinase powder were optimum, at 1,150 ± 40 IU/ml and 95 ± 3, respectively. The moisture content of the nattokinase powder was 4.4 ± 0.2%. According to the nattokinase activity, comprehensive score, and moisture content of nattokinase powder, the scopes of 25%–35% mass ratios of wall materials were chosen for further experiments.

### 3.2 Effect of inlet air temperature on spray drying of nattokinase powder

Heated air, usually used as a heat source, has an important influence on the drying effect and the final product features. If the inlet air temperature is too low, the moisture of the small droplets cannot be fully evaporated, which would affect the product quality; if the inlet air temperature is too high, the enzyme activity will be decreased (Zhang et al., 2019). The mass ratio of wall materials was set at 30%, the feed rate at 10 L/h, and the outlet temperature at 90°C. Spray drying of the nattokinase powder was carried out under the conditions of inlet air temperature of 120, 130, 140, 150, and 160°C, respectively (Figure 1b).

When the inlet air temperatures were increased from 120–140°C, nattokinase activity (*p* < .05) and comprehensive score (*p* < .05) of the nattokinase powder increased significantly (Figure 1b). Nattokinase activity and comprehensive score of the nattokinase powder reached the highest when the inlet air temperature was 140°C, as 1,178 ± 20 IU/ml and 95 ± 3, respectively. The moisture content of the nattokinase powder was 4.3 ± 0.1%. During the spray drying process, the nattokinase powder at the inlet air temperature of 140°C was white or light yellow and had a unique odor, and the dispersion of the nattokinase powder was best. When the inlet air temperature was too already high, the residual sugar in the fermentation broth would undergo Maillard reaction between the proteins with carbohydrates to produce brown substance, which had a paste smell (Hamdi et al., 2020; Huang et al., 2020). Although the high inlet air temperature helps to reduce the moisture content, the spray‐dried nattokinase powder by microencapsulation was easy to agglomerate and reduced product quality and nattokinase activity. According to activity, comprehensive score and moisture content of the nattokinase powder, the scopes of 130–150°C of inlet air temperature were chosen for further experiments.

### 3.3 Effect of feed rate on spray drying of nattokinase powder

If the feed rate is already too high, inadequate product would be produced, like as high moisture content (Ziaee et al., 2020). The mass ratio of wall materials was set at 30%, the inlet air temperature at 140°C, and the outlet temperature at 90°C. Spray drying of the nattokinase powder was carried out under the conditions of feed rates of 4, 6, 8, 10, and 12 L/h, respectively (Figure 1c).

Nattokinase activities and comprehensive scores of the nattokinase powder increased first and then decreased with the increase of the feed rates of 4–12 L/h (Figure 1c). When the feed rate was 8 L/h, nattokinase activity and comprehensive score of the nattokinase powder were optimum, at 1,172 ± 23 IU/ml and 93 ± 1, respectively. The moisture content of the nattokinase powder was 4.2 ± 0.2%. When the feed rate was too high, it caused wall sticking and high moisture content during the spray drying process, and the nattokinase powder had peculiar and paste smell, and the color was burnt brown. According to nattokinase activity, comprehensive score, and moisture content of the nattokinase powder, the scopes of 6–10 L/h of feed rate were chosen for further experiments.

### 3.4 Effect of outlet temperature on spray drying of nattokinase powder

Spray drying is a high‐temperature short time process. Cautious control of the outlet temperature can hold back the bioactivity of functional factors of interest. Inactivation of enzymes would rise as the outlet temperatures increase and decrease as the outlet temperature decrease (Lavanya et al., 2020). The mass ratio of wall materials was set at 30%, the inlet air temperature at 140°C, and the feed rate at 8 L/h. Spray drying of the nattokinase powder was carried out under the conditions of outlet temperature of 60, 70, 80, 90, and 100°C, respectively (Figure 1d).

Nattokinase activities and comprehensive scores of the nattokinase powder increased first and then decreased with the increase of the outlet temperature of 60–100°C (Figure 1d). When the outlet temperature was 80°C, the comprehensive score was optimum, as 95 ± 1. However, optimal outlet temperature of nattokinase activity was 90°C; the nattokinase activity was 1,190 ± 24 IU/ml. The moisture content of the nattokinase powder was 4.2 ± 0.1%. When the outlet temperature was 80–90°C, the nattokinase powder was white or light yellow and had a unique flavor. When the outlet temperature was low, the nattokinase powder was easy to stick to the wall and agglomerate and had a high‐moisture content. In addition, the high outlet temperature caused the nattokinase activity to decrease. According to nattokinase activities, comprehensive scores and moisture content of the nattokinase powder, the scopes of 70–90°C of outlet temperature were chosen for further experiments.

### 3.5 RSM results on spray drying of nattokinase powder

Box‐Benkhen experimental design has been applied for the optimization of the various processing parameters and their interactions (Gil‐Chávez et al., 2020; Hayta^1^& Ertop, 2017; Slima et al., 2020). Based on the above single‐factor experimental results, the Box‐Behnken experimental design was used according to the selected parameters of mass ratio of wall materials, inlet air temperature, feed rate, and outlet temperature. The factors, levels, and results of the RSM experiment are shown in Table S1.

A multiple quadratic regression model was built for four significant influence processing parameters of the comprehensive scores (*Y*_1_) of the nattokinase powder, like as mass ratio of wall materials (*A*), inlet air temperature (*B*), feed rate (*C*), and outlet temperature (*D*), using Design‐Expert 8.0 software (Stat‐Ease, USA). The obtained quadratic regression Equation (2) was as following:

(2) $$Y_{1}=96.80+5.08A‐0.75B‐0.083C+1.08D+1.50AB‐0.50AC+0.25AD+0.25BC+1.00BD‐1.50CD‐6.78A^{2}‐3.53B^{2}‐3.27C^{2}‐3.02D^{2}$$

Results of ANOVA of the comprehensive scores of the nattokinase powder are shown in Table S2. The Model *F*‐value of 187.91 implied the model is significant. Moreover, the *R* ^2^ was 0.9947 and Adj *R* ^2^ was 0.9894, indicating that the model‐fitting degree was well and only 0.53% of the variation could not be attributed to this model. Overall, this model could be utilized for the reliable prediction of experiment results. The lack of fit was 0.7512, which was more than 0.05, indicating that the lack of fit was nonsignificant relative to the pure error in this experiment (Ma et al., 2020; Qiu et al., 2016). The mass ratio of wall materials, inlet air temperature, and outlet temperature significantly affected the comprehensive scores of the nattokinase powder in this experiment. The orders of influencing factors on the comprehensive scores of the nattokinase powder were as follows: mass ratio of wall materials >outlet temperature >inlet air temperature >feed rate.

**FIGURE 2:**
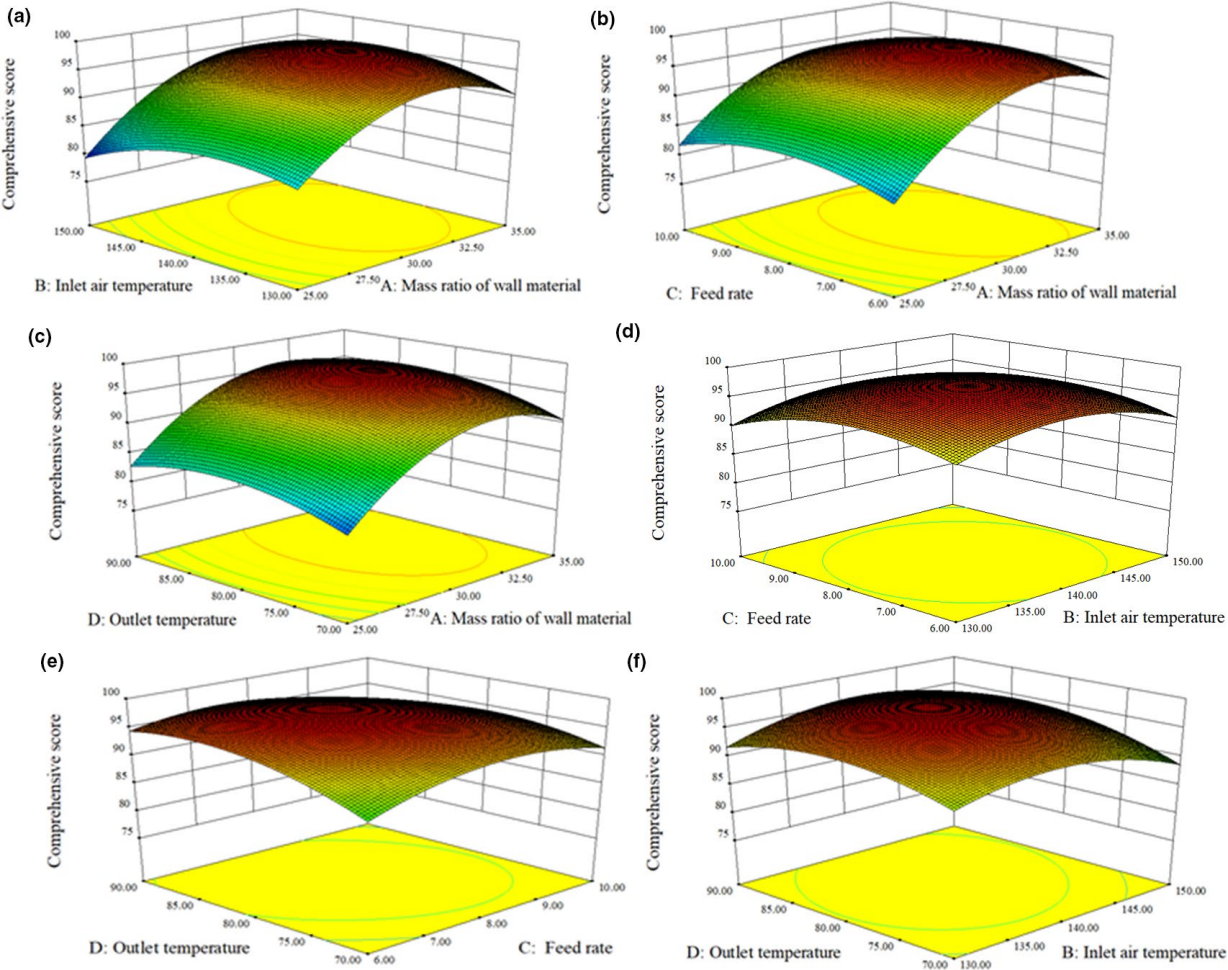
Effects of the interaction of different factors on the comprehensive scores of nattokinase powder by microencapsulation: (a) mass ratio of wall material and inlet air temperature; (b) mass ratio of wall material and feed rate; (c) mass ratio of wall material and outlet temperature; (d) inlet air temperature and fees rate; (e) feed rate and outlet temperature; (f) inlet air temperature and outlet temperature

Results of Figure 2 showed that with the increase of the mass ratio of wall materials, inlet air temperature, feed rate, and outlet temperature, the comprehensive scores of the nattokinase powder first increased and then decreased, and its change trends were consistent with the single‐factor experimental results. According to this model, the optimal conditions were as follows: The mass ratio of wall materials 32.40%, inlet air temperature 138.9°C, feed rate 7.98 L/h, and outlet temperature 81.2°C. Considering the actual operation, the process conditions were modified as follows: The mass ratio of wall materials 32%, air inlet temperature 139°C, feed rate 8 L/h, outlet temperature 81°C. The results showed that the microencapsulated nattokinase powder samples were light yellow, with a special smell, no peculiar smell and paste taste, and no impurity. The average moisture content of nattokinase powder was 4.2 ± 0.1%, the average nattokinase activity was 1,320 ± 40 IU/ml, and an average comprehensive score was 97 ± 1, which were consistent with the predicted values.

Similar to the comprehensive scores of the nattokinase powder, a multiple quadratic response surface regression model was also established for the nattokinase activities (*Y_2_*) of the microencapsulated nattokinase powder. The obtained quadratic regression Equation (3) was as following:

(3) $$Y_{2}=‐47568.00+528.17A+402.48B+440.75C+278.33D+0.56AB‐0.58AC‐0.31AD+1.86BC+0.12BD‐0.83CD‐9.42A^{2}‐1.59B^{2}‐38.46C^{2}‐1.75D^{2}$$

Results of ANOVA of the nattokinase activities are shown in Table S3. The *F* test of the regression model presented high significance (*p* < .001), the *R* ^2^ was 0.9794 and Adj *R* ^2^ was 0.9588, indicating that the fitting degree of this model was good, and only 2.16% of the variation could not be predicted. The lack of fit was 0.4396 more than 0.05, which was nonsignificant relative to the pure error. The mass ratio of wall materials, inlet air temperature, and outlet temperature significantly affected the nattokinase activities of the nattokinase powder in this experiment. The orders of influencing factors on nattokinase activities of the nattokinase powder were as follows: inlet air temperature >mass ratio of wall materials >outlet temperature >feed rate.

**FIGURE 3:**
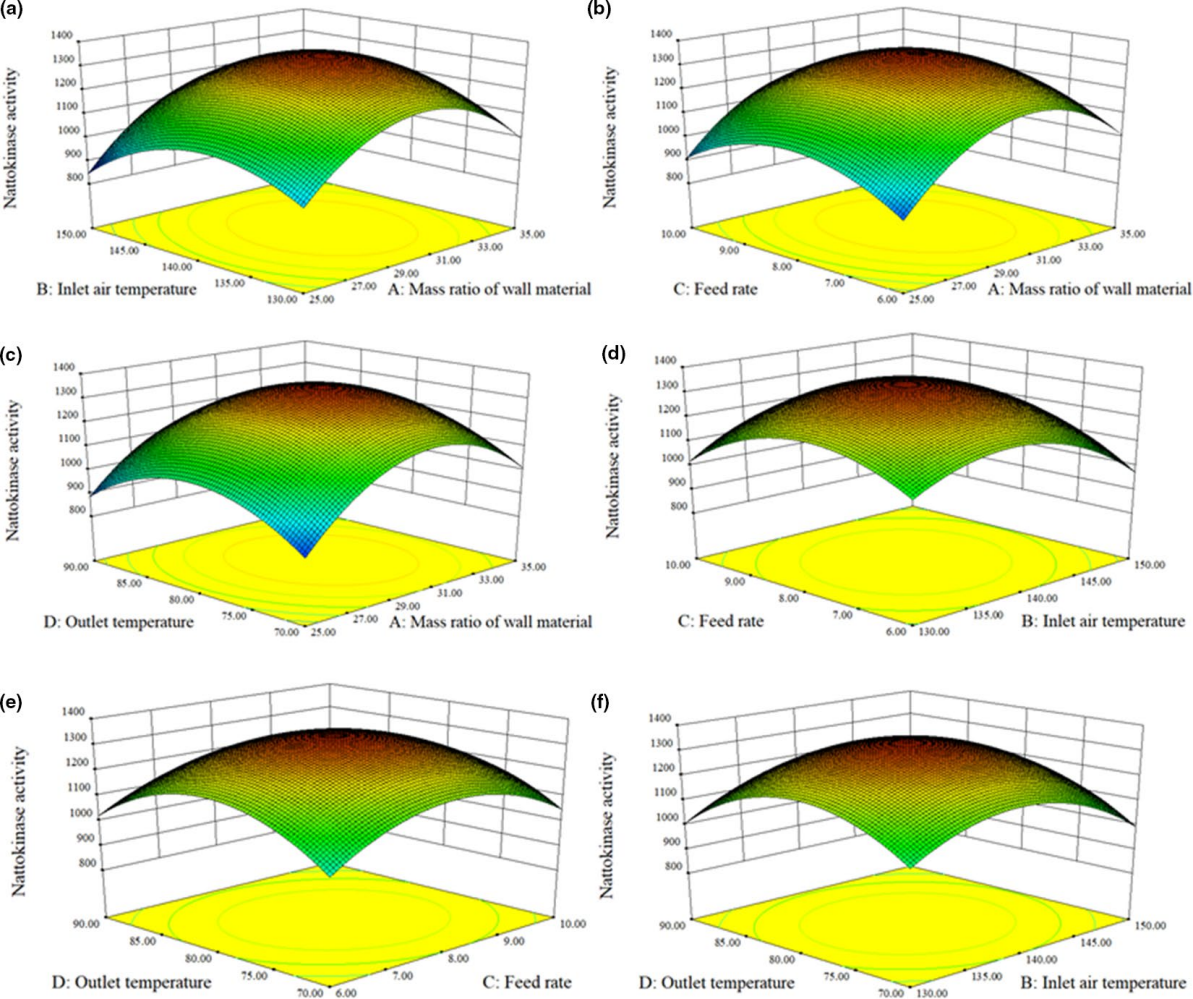
Effects of the interaction of different factors on the nattokinase activities of nattokinase powder by microencapsulation: (a) mass ratio of wall material and inlet air temperature; (b) mass ratio of wall material and feed rate; (c) mass ratio of wall material and outlet temperature; (d) inlet air temperature and feed rate; (e) feed rate and outlet temperature; (f) inlet air temperature and outlet temperature

Results of Figure 3 showed that with the increase of the mass ratio of wall materials, inlet air temperature, feed rate, and outlet temperature, the nattokinase activities of the nattokinase powder first increased and then decreased, and its change trends were consistent with the single‐factor experimental results. According to this model, the optimal conditions were as follows: The mass ratio of wall materials 30.58%, inlet air temperature 139.49°C, feed rate 8.02 L/h, and outlet temperature 79.71°C. Considering the actual operation, the process conditions were modified as follows: The mass ratio of wall materials 30%, air inlet temperature 139°C, feed rate 8 L/h, and outlet temperature 80°C. The results showed that the nattokinase powder was light yellow, with a special smell, no peculiar smell and paste taste, and no impurity. The average moisture content of microencapsulated nattokinase powder was 4.1 ± 0.1%, the average nattokinase activity was 1,340 ± 45 IU/ml, and the average comprehensive score was 96 ± 2, which were consistent with the predicted values. According to the results of RSM experiments described above, the optimal conditions of nattokinase activities and comprehensive scores were virtually identical.

### 3.6 Storage stability in different temperature or different pH

**FIGURE 4:**
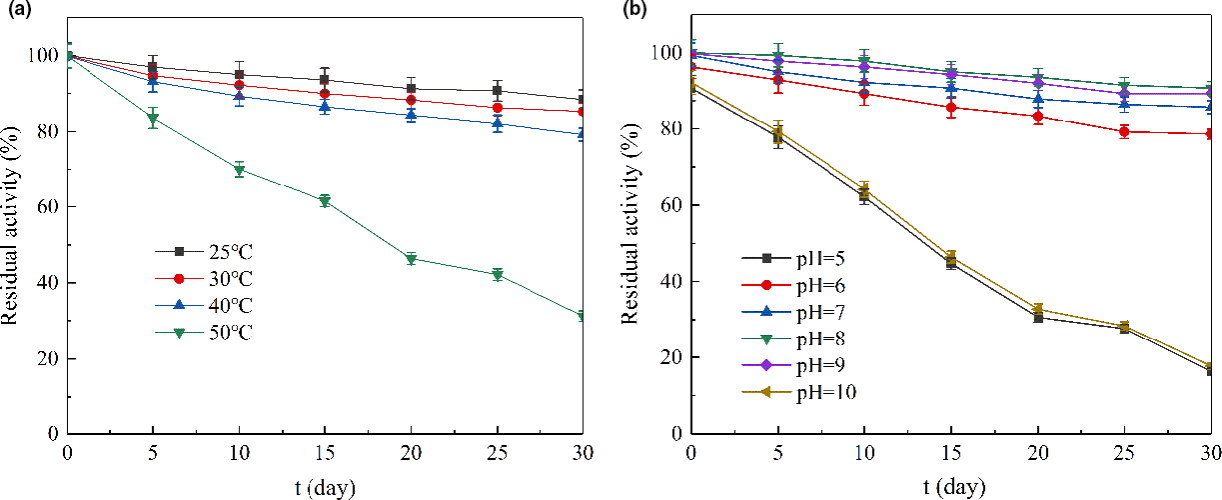
Storage stability of nattokinase powder by microencapsulation in different temperature (a) or different pH (b)

Storage stability is the key to evaluate the market value and practical application value of functional foods (Kumar et al., 2020; Tolun et al., 2016; Xiang et al., 2020) or functional strains (Baena‐Aristizábal et al., 2019). Functional foods that cannot be stored stably for a long time under harsh conditions are resistant to profitable marketing, especially the functional factors of enzymes. Therefore, the storage stability of the microencapsulated nattokinase powder was tested under different temperatures (25, 30, 40, and 50°C) and pH (5, 6, 7, 8, 9, and 10) conditions for the 30 days (Figure 4).

The storage temperature has a significant effect on activity, color, and smell (Christiansen et al., 2020; Guo et al., 2020). Elevated storage temperature easily reduces the activity, while low storage temperature increases the cost. Microencapsulated nattokinase powder samples were stable at 25, 30, and 40°C after 30 days of storage, the nattokinase activities of the nattokinase powder still kept 88.5, 85.3, and 80.0% of the initial activity respectively (Figure 4a). When the storage temperature was above 50°C, the nattokinase activity of nattokinase powder decreased rapidly. Nattokinase microencapsulated in carbohydrates which significantly enhanced storage stability can be stored at room temperature for a long time. Wall materials can protect enzyme molecules by providing stable microenvironment and reduce the damage caused by harsh environment. The wall materials of whey proteins with gum Arabic or maltodextrin increased the polyphenols stability and their antioxidant capacity during storage (Khalifa et al., 2019). After 30 days of storage at 25°C, the microencapsulated nattokinase powder was still light yellow, with a special smell, no peculiar smell and paste taste, and no impurity.

Storage pH significantly affected the activity and content of functional factors (Bradwell et al., 2018; Liu et al., 2020). Storage stability of the nattokinase powder was better when pH was 6–9; the storage stability of the nattokinase powder with pH 8 was the best. After 30 days of storage, the activity of the microencapsulated nattokinase powder still kept 90.8% of the initial activity at pH 8 (Figure 4b). When the storage pH was 5 and 10, the activities of the nattokinase powder decreased rapidly. Microencapsulated nattokinase showed strong storage stability in different storage pH. Nattokinase powder can be kept at room temperature and near‐neutral pH for a long time. After 30 days of storage at 25°C and pH 8.0, the microencapsulated nattokinase powder was still white or light yellow, with a special smell, no peculiar smell and paste taste, and no impurity.

## 4 CONCLUSION

In this work, the effects of four different critical parameters, namely the mass ratio of wall materials, inlet air temperature, feed rate, and outlet temperature, on the spray drying of the microencapsulated nattokinase powder were studied in the single‐factor experiments and RSM experiments. Two quadratic polynomial regression models were established with the comprehensive scores and nattokinase activities as the response values. The results showed that Box‐Behnken CCD of RSM is feasible and effective for the optimization of the spray drying microencapsulation process of nattokinase powder. According to the single‐factor experiments and RSM analysis, optimized spray drying process parameters were 30% of mass ratio of wall materials, 139°C of air inlet temperature, 8 L/h of feed rate, and 80°C of outlet temperature. The final optimized results encompassed a comprehensive score of 96 and nattokinase activity of 1,340 IU/ml. Microencapsulated nattokinase showed strong storage stability in different storage temperature and pH. Nattokinase powder can be stored at room temperature and near‐neutral pH for a long time. Overall, these results provide a foundation for further green industrialization of the nattokinase powder from fermentation broth.

## CONFLICT OF INTEREST

There is no conflict of interest in this work.

## Supporting information

App S1

## References

[fsn32378-bib-0001] Alcântara, M. A. , de Lima, A. E. A. , Mattos Braga, A. L. , Tonon, R. V. , Galdeano, M. C. , da Costa Mattos, M. , Brígida, A. I. S. , Rosenhaim, R. , dos Santos, N. A. , & de Magalhães Cordeiro, A. M. T. (2019). Influence of the emulsion homogenization method on the stability of chia oil microencapsulated by spray drying. Powder Technology, 354, 877–885. 10.1016/j.powtec.2019.06.026

[fsn32378-bib-0002] Amin, K. , Zeng, X. , You, Y. , Hu, Y. , Sun, H. , Lyu, B. , Piao, C. , & Yu, H. (2020). Enhanced thermostability and antioxidant activity of nattokinase by biogenic enrichment of selenium. Journal of Food Measurement and Characterization, 14, 2145–2154. 10.1007/s11694-020-00461-w

[fsn32378-bib-0003] Baena‐Aristizábal, C. M. , Foxwell, M. , Wright, D. , & Villamizar‐Rivero, L. (2019). Microencapsulation of *Rhizobium* *leguminosarum* bv. *trifolii* with guar gum: Preliminary approach using spray drying. Journal of Biotechnology, 302, 32–41. 10.1016/j.jbiotec.2019.06.007 31201836

[fsn32378-bib-0004] Bajaj, S. R. , Marathe, S. J. , & Singhal, R. S. (2021). Co‐encapsulation of vitamins B_12_ and D_3_ using spray drying: Wall material optimization, product characterization, and release kinetics. Food Chemistry, 335, 127642. 10.1016/j.foodchem.2020.127642 32739814

[fsn32378-bib-0005] Bamidele, O. P. , & Emmambux, M. N. (2019). Storage stability of encapsulated ascorbyl palmitate in normal and high amylose maize starches during pasting and spray drying. Carbohydrate Polymers, 216, 217–223. 10.1016/j.carbpol.2019.04.022 31047060

[fsn32378-bib-0006] Bilušić, T. , Drvenica, I. , Kalušević, A. , Marijanović, Z. , Jerković, I. , Mužek, M. N. , Bratanić, A. , Skroza, D. , Zorić, Z. , Pedisić, S. , Nedović, V. , & Režek Jambrak, A. (2021). Influences of freeze‐ and spray‐drying vs. encapsulation with soy and whey proteins on gastrointestinal stability and antioxidant activity of Mediterranean aromatic herbs. International Journal of Food Science & Technology, 56(4), 1582–1596. 10.1111/ijfs.14774

[fsn32378-bib-0007] Bradwell, J. , Hurd, M. , Pangloli, P. , McClure, A. , & Dia, V. P. (2018). Storage stability of sorghum phenolic extracts' flavones luteolin and apigenin. LWT ‐ Food Science and Technology, 97, 787–793. 10.1016/j.lwt.2018.08.006

[fsn32378-bib-0008] Bustamante, M. , Laurie‐Martínez, L. , Vergara, D. , Campos‐Vega, R. , Rubilar, M. , & Shene, C. (2020). Effect of three polysaccharides (inulin, and mucilage from chia and flax seeds) on the survival of probiotic bacteria encapsulated by spray drying. Applied Sciences, 10, 4623. 10.3390/app10134623

[fsn32378-bib-0009] Cai, D. , Zhu, C. , & Chen, S. (2017). Microbial production of nattokinase: Current progress, challenge and prospect. World Journal of Microbiology & Biotechnology, 33, 84. 10.1007/s11274-017-2253-2 28378222

[fsn32378-bib-0010] Chávez‐Salazar, A. , Castellanos‐Galeano, F. J. , Álvarez‐Barreto, C. I. , Bello‐Pérez, L. A. , Cortés‐Rodríguez, M. , & Hoyos‐Leyva, J. D. (2019). Optimization of the spray drying process of the esterified plantain starch by response surface methodology. Starch ‐ Stärke, 71, 1800330. 10.1002/star.201800330

[fsn32378-bib-0011] Chen, C. , Duan, H. , Gao, C. , Liu, M. , Wu, X. , Wei, Y. , Zhang, X. , & Liu, Z. (2014). Non‐covalent modification of thrombolytic agent nattokinase: Simultaneous improvement of fibrinolysis activity and enzymatic stability. RSC Advance, 4, 27422–27429. 10.1039/C4RA02626H

[fsn32378-bib-0012] Chen, F. , Liu, L. , & Tang, C. (2020). Spray‐drying microencapsulation of curcumin nanocomplexes with soy protein isolate: Encapsulation, water dispersion, bioaccessibility and bioactivities of curcumin. Food Hydrocolloids, 105, 105821. 10.1016/j.foodhyd.2020.105821

[fsn32378-bib-0013] Christiansen, M. V. , Pedersen, T. B. , Brønd, J. N. , Skibsted, L. H. , & Ahrné, L. (2020). Physical properties and storage stability of reverse osmosis skim milk concentrates: Effects of skim milk pasteurisation, solid content and thermal treatment. Journal of Food Engineering, 278, 109922. 10.1016/j.jfoodeng.2020.109922

[fsn32378-bib-0014] Devi, C. S. , Mohanasrinivasan, V. , Sharma, P. , Das, D. , Vaishnavi, B. , & Naine, S. J. (2016). Production, purification and stability studies on nattokinase: A therapeutic protein extracted from mutant *Pseudomonas aeruginosa* CMSS isolated from bovine milk. International Journal of Peptide Research and Therapeutics, 22, 263–269. 10.1007/s10989-015-9505-5

[fsn32378-bib-0015] Ding, Z. , Tao, T. , Yin, X. , Prakash, S. , Wang, X. , Zhao, Y. , Han, J. , & Wang, Z. (2020). Improved encapsulation efficiency and storage stability of spray dried microencapsulated lutein with carbohydrates combinations as encapsulating material. LWT ‐ Food Science and Technology, 124, 109139. 10.1016/j.lwt.2020.109139

[fsn32378-bib-0016] Garg, R. , & Thorat, B. N. (2014). Nattokinase purification by three phase partitioning and impact of t‐butanol on freeze drying. Separation and Purification Technology, 131, 19–26. 10.1016/j.seppur.2014.04.011

[fsn32378-bib-0017] Gil‐Chávez, J. , Padhi, S. S. P. , Hartge, U. , Heinrich, S. , & Smirnova, I. (2020). Optimization of the spray‐drying process for developing aquasolv lignin particles using response surface methodology. Advanced Powder Technology, 31, 2348–2356. 10.1016/j.apt.2020.03.027

[fsn32378-bib-0018] González, F. , García‐Martínez, E. , del Mar Camacho, M. , & Martínez‐Navarrete, N. (2019). Stability of the physical properties, bioactive compounds and antioxidant capacity of spray‐dried grapefruit powder. Food Bioscience, 28, 74–82. 10.1016/j.fbio.2019.01.009

[fsn32378-bib-0019] Guo, X. , Wu, S. , & Zhu, K. (2020). Effect of superheated steam treatment on quality characteristics of whole wheat flour and storage stability of semi‐dried whole wheat noodle. Food Chemistry, 322, 126738. 10.1016/j.foodchem.2020.126738 32283361

[fsn32378-bib-0020] Hamdi, M. , Nasri, R. , Azaza, Y. B. , Li, S. , & Nasri, M. (2020). Conception of novel blue crab chitosan films crosslinked with different saccharides *via* the Maillard reaction with improved functional and biological properties. Carbohydrate Polymers, 241, 116303. 10.1016/j.carbpol.2020.116303 32507187

[fsn32378-bib-0021] Hayta, M. , & Ertop, M. H. (2017). Optimisation of sourdough bread incorporation into wheat breadby response surface methodology: Bioactive and nutritionalproperties. International Journal of Food Science and Technology, 52, 1828–1835. 10.1111/ijfs.13457

[fsn32378-bib-0022] Hoskin, R. T. , Xiong, J. , Esposito, D. A. , & Lila, M. A. (2019). Blueberry polyphenol‐protein food ingredients: The impact of spray drying on the in vitro antioxidant activity, anti‐inflammatory markers, glucose metabolism and fibroblast migration. Food Chemistry, 280, 187–194. 10.1016/j.foodchem.2018.12.046 30642485

[fsn32378-bib-0023] Huang, G. , Wang, H. , Wang, F. , Du, Y. , & Xiao, J. (2020). Maillard reaction in protein – polysaccharide coacervated microcapsules and its effects on microcapsule properties. International Journal of Biological Macromolecules, 155, 1194–1201. 10.1016/j.ijbiomac.2019.11.087 31726167

[fsn32378-bib-0024] Huang, L. X. , & Mujumdar, A. S. (2008). The effect of rotary disk atomizer RPM on particle size distribution in a semi‐industrial spray dryer. Drying Technology, 26, 1319–1325. 10.1080/07373930802330938

[fsn32378-bib-0025] Khalifa, I. , Li, M. , Mamet, T. , & Li, C. (2019). Maltodextrin or gum Arabic with whey proteins as wall‐material blends increased the stability and physiochemical characteristics of mulberry microparticles. Food Bioscience, 31, 100445. 10.1016/j.fbio.2019.100445

[fsn32378-bib-0026] Kumar, S. S. , Chauhan, A. S. , & Giridhar, P. (2020). Nanoliposomal encapsulation mediated enhancement of betalain stability: Characterisation, storage stability and antioxidant activity of *Basella rubra* L.fruits for its applications in vegan gummy candies. Food Chemistry, 333, 127442. 32673950 10.1016/j.foodchem.2020.127442

[fsn32378-bib-0027] Kurosawa, Y. , Nirengi, S. , Homma, T. , Esaki, K. , Ohta, M. , Clark, J. F. , & Hamaoka, T. (2015). A single‐dose of oral nattokinase potentiates thrombolysis and anticoagulation profiles. Scientific Reports, 5, 11601. 10.1038/srep11601 26109079 PMC4479826

[fsn32378-bib-0028] Lavanya, M. N. , Kathiravan, T. , Moses, J. A. , & Anandharamakrishnan, C. (2020). Influence of spray‐drying conditions on microencapsulation of fish oil and chia oil. Drying Technology, 38, 279–292. 10.1080/07373937.2018.1553181

[fsn32378-bib-0029] Liu, J. , Mu, T. , Sun, H. , & Fauconnier, M. L. (2020). Effects of processing and storage conditions on the stability of sweet potato (*Ipomoea* *batatas* L.) leaf flavonoids. International Journal of Food Science and Technology, 55, 2251–2260.

[fsn32378-bib-0030] Liu, Z. , Zhao, H. , Han, L. , Cui, W. , Zhou, L. , & Zhou, Z. (2019). Improvement of the acid resistance, catalytic efficiency, and thermostability of nattokinase by multisite‐directed mutagenesis. Biotechnology and Bioengineering, 116, 1833–1843. 10.1002/bit.26983 30934114

[fsn32378-bib-0031] Ma, W. , Zhang, J. , Shu, L. , Tan, Z. , An, Y. , Yang, X. , Wang, D. , & Gao, Q. (2020). Optimization of spray drying conditions for the green manufacture of γ‐ aminobutyric acid‐rich powder from *Lactobacillus brevis* fermentation broth. Biochemical Engineering Journal, 156, 107499. 10.1016/j.bej.2020.107499

[fsn32378-bib-0032] Man, L. , Xiang, D. , & Zhang, C. (2019). Strain screening from traditional fermented soybean foods and induction of nattokinase production in *Bacillus subtilis* MX‐6. Probiotics and Antimicrobial Proteins, 11, 283–294. 10.1007/s12602-017-9382-7 29411244

[fsn32378-bib-0033] Ni, H. , Guo, P. , Jiang, W. , Fan, X. , Luo, X. , & Li, H. (2016). Expression of nattokinase in *Escherichia coli* and renaturation of itsinclusion body. Journal of Biotechnology, 231, 65–71. 10.1016/j.jbiotec.2016.05.034 27234878

[fsn32378-bib-0034] Obón, J. M. , Luna‐Abad, J. P. , Bermejo, B. , & Fernández‐López, J. A. (2020). Thermographic studies of cocurrent and mixed flow spray drying of heat sensitive bioactive compounds. Journal of Food Engineering, 268, 109745. 10.1016/j.jfoodeng.2019.109745

[fsn32378-bib-0035] Qiu, J. , Zhang, H. , Wang, Z. , Liu, S. , & Regenstein, J. M. (2016). Response surface methodology for the synthesis of an *Auricularia auriculajudae* polysaccharides‐CDDP complex. International Journal of Biological Macromolecules, 93, 333–343. 10.1016/j.ijbiomac.2016.06.066 27343707

[fsn32378-bib-0036] Rezvankhah, A. , Emam‐Djomeh, Z. , & Askari, G. (2020). Encapsulation and delivery of bioactive compounds using spray and freeze‐drying techniques: A review. Drying Technology, 38, 235–258. 10.1080/07373937.2019.1653906

[fsn32378-bib-0037] Slima, S. B. , Trabelsi, I. , Ktari, N. , Kriaa, M. , Abdeslam, A. , Herrero, A. M. , Jiménez‐Colmenero, F. , Ruiz‐Capillas, C. , & Salah, R. B. (2020). Modeling the influence of functional additives in beef sausages using a Box‐Benkhen design: Effects on quality characteristics. Food Bioscience, 35, 100572.

[fsn32378-bib-0038] Tolun, A. , Altintas, Z. , & Artik, N. (2016). Microencapsulation of grape polyphenols using maltodextrin and gum arabic as two alternative coating materials: Development and characterization. Journal of Biotechnology, 239, 23–33. 10.1016/j.jbiotec.2016.10.001 27720817

[fsn32378-bib-0039] Wang, Y. , & Selomulya, C. (2020). Spray drying strategy for encapsulation of bioactive peptide powders for food applications. Advanced Powder Technology, 31, 409–415. 10.1016/j.apt.2019.10.034

[fsn32378-bib-0040] Weng, Y. , Yao, J. , Sparks, S. , & Wang, K. Y. (2017). Nattokinase: An oral antithrombotic agent for the prevention of cardiovascular disease. International Journal of Molecular Sciences, 18, 523. 10.3390/ijms18030523 PMC5372539 28264497

[fsn32378-bib-0041] Xiang, C. , Gao, J. , Ye, H. , Ren, G. , Ma, X. , Xie, H. , Fang, S. , Lei, Q. , & Fang, W. (2020). Development of ovalbumin‐pectin nanocomplexes for vitamin D_3_ encapsulation: Enhanced storage stability and sustained release in simulated gastrointestinal digestion. Food Hydrocolloids, 106, 105926. 10.1016/j.foodhyd.2020.105926

[fsn32378-bib-0042] Yingngam, B. , Kacha, W. , Rungseevijitprapa, W. , Sudta, P. , Prasitpuriprecha, C. , & Brantner, A. (2019). Response surface optimization of spray‐dried citronella oil microcapsules with reduced volatility and irritation for cosmetic textile uses. Powder Technology, 355, 372–385. 10.1016/j.powtec.2019.07.065

[fsn32378-bib-0043] Zhang, S. , Lei, H. , Gao, X. , Xiong, X. , Wu, W. D. , Wu, Z. , & Chen, X. D. (2018). Fabrication of uniform enzyme‐immobilized carbohydrate microparticles with high enzymatic activity and stability *via* spray drying and spray freeze drying. Powder Technology, 330, 40–49. 10.1016/j.powtec.2018.02.020

[fsn32378-bib-0044] Zhang, Z. , Peng, H. , Ma, H. , & Zeng, X. (2019). Effect of inlet air drying temperatures on the physicochemical properties and antioxidant activity of whey protein isolate‐kale leaves chlorophyll (WPI‐CH) microcapsules. Journal of Food Engineering, 245, 149–156. 10.1016/j.jfoodeng.2018.10.011

[fsn32378-bib-0045] Ziaee, A. , Albadarin, A. B. , Padrela, L. , Ung, M. , Femmer, T. , Walker, G. , & O'Reilly, E. (2020). A rational approach towards spray drying of biopharmaceuticals: The case of lysozyme. Powder Technology, 366, 206–215. 10.1016/j.powtec.2020.02.057

