# Supplementary material for: Microencapsulation of nattokinase from fermentation by spray drying: Optimization, comprehensive score, and stability
Source: Food Sci Nutr. 2021 Jun 8;9(7):3906–16. doi: 10.1002/fsn3.2378 (PMC8269611; doi:10.1002/fsn3.2378)
Supplement: Supplementary file 1 — App S1 [file FSN3-9-3906-s001.docx]

**Appendix S1**

**Supporting Information**

**Microencapsulation of nattokinase from fermentation by spray drying: Optimization, comprehensive score and stability**

Ganlu Li ^1^, Tao Li ^1^, Feng He ^2^, Cheng Chen ^1^, Xu Xu ^1^, Weilong Tian ^2^, Yue Yang ^1^, Xun He ^1^, Hui Li ^1,*^, Kequan Chen ^1^, Ning Hao ^1^, Pingkai Ouyang ^1^

^1^ College of Biotechnology and Pharmaceutical Engineering, Nanjing Tech University, Nanjing, 211816, China

^2^ Jiangsu Jicui Industrial Biotechnology Research Institute Co., Ltd, Nanjing, 210000, China

*^*^*Corresponding author. E-mail: lihui11@njtech.edu.cn

**Table S1** The factors and levels from the RSM analysis experiment

| Experiment number | A/Mass ratio of wall material (%) | B/Inlet air temperature (℃) | C/Feed rate (L/h) | D/Outlet temperature (℃) | Y_1_/Comprehensive score | Y_2_/ Nattokinase activity (IU/mL) |
| --- | --- | --- | --- | --- | --- | --- |
| 1 | 1 | 0 | 0 | 1 | 94 | 1014 |
| 2 | 1 | 1 | 0 | 0 | 92 | 995 |
| 3 | -1 | -1 | 0 | 0 | 84 | 956 |
| 4 | 0 | 0 | 0 | 0 | 97 | 1369 |
| 5 | 0 | 0 | 0 | 0 | 97 | 1358 |
| 6 | -1 | 0 | 0 | -1 | 81 | 836 |
| 7 | 1 | 0 | 0 | -1 | 91 | 1008 |
| 8 | 0 | 0 | 1 | 1 | 90 | 996 |
| 9 | 0 | 0 | 0 | 0 | 97 | 1349 |
| 10 | -1 | 1 | 0 | 0 | 79 | 854 |
| 11 | 0 | 1 | 0 | -1 | 87 | 987 |
| 12 | 1 | -1 | 0 | 0 | 91 | 986 |
| 13 | 0 | 1 | 1 | 0 | 90 | 1046 |
| 14 | 0 | -1 | 0 | -1 | 90 | 1046 |
| 15 | 0 | -1 | -1 | 0 | 91 | 1105 |
| 16 | 0 | 0 | 0 | 0 | 97 | 1296 |
| 17 | 0 | 0 | -1 | 1 | 93 | 974 |
| 18 | 0 | -1 | 0 | 1 | 91 | 996 |
| 19 | 0 | 1 | -1 | 0 | 95 | 994 |
| 20 | 1 | 0 | 1 | 0 | 91 | 997 |
| 21 | -1 | 0 | 0 | 1 | 83 | 904 |
| 22 | 0 | 0 | -1 | -1 | 88 | 998 |
| 23 | 0 | 0 | 1 | -1 | 91 | 1086 |
| 24 | -1 | 0 | 1 | 0 | 82 | 903 |
| 25 | -1 | 0 | -1 | 0 | 81 | 889 |
| 26 | 0 | -1 | 1 | 0 | 90 | 1008 |
| 27 | 1 | 0 | -1 | 0 | 92 | 1106 |
| 28 | 0 | 1 | 0 | 1 | 91 | 986 |
| 29 | 0 | 0 | 0 | 0 | 96 | 1348 |

Note: each experiment was repeated 3 times, and the average value was taken.

**Table S2** Regression analysis for the comprehensive scores according to the Box-Behnken experimental design

| Source | SS | DF | MS | F value | *p* | Significance |
| --- | --- | --- | --- | --- | --- | --- |
| Model | 714.06 | 14 | 51.72 | 187.91 | <0.0001 | *** |
| *A* | 310.08 | 1 | 310.08 | 1142.41 | <0.0001 | *** |
| *B* | 6.75 | 1 | 6.75 | 24.81 | 0.0002 | ** |
| *C* | 0.083 | 1 | 0.083 | 0.31 | 0.5883 |  |
| *D* | 14.08 | 1 | 14.08 | 51.89 | <0.0001 | *** |
| *AB* | 9.00 | 1 | 9.00 | 33.16 | <0.0001 | *** |
| *AC* | 1.00 | 1 | 1.00 | 3.68 | 0.0755 |  |
| *AD* | 0.25 | 1 | 0.25 | 0.92 | 0.3535 |  |
| *BC* | 0.25 | 1 | 0.25 | 0.92 | 0.0018 | ** |
| *BD* | 4.00 | 1 | 4.00 | 14.74 | <0.0001 | *** |
| *CD* | 9.00 | 1 | 9.00 | 33.16 | <0.0001 | *** |
| *A^2^* | 297.73 | 1 | 297.73 | 1096.91 | <0.0001 | *** |
| *B^2^* | 80.60 | 1 | 80.60 | 296.94 | <0.0001 | *** |
| *C^2^* | 69.57 | 1 | 69.57 | 256.32 | <0.0001 | *** |
| *D^2^* | 59.36 | 1 | 59.36 | 218.68 | <0.0001 | *** |
| Residual | 3.80 | 14 | 0.27 |  |  |  |
| Lack of fit | 3.00 | 10 | 0.30 | 0.63 | 0.7512 |  |
| Pure error | 0.80 | 4 | 0.20 |  |  |  |
| Cor total | 717.86 | 28 |  |  |  |  |

Note：^*^ *p* < 0.05；^**^ *p* < 0.01；^***^*p* < 0.001

*R*^2^ = 0.9947, Adj*R*^2^ = 0.9894,

SS: Sum of squares; DF: Degree freedom; MS: Mean square.

**Table S3** Regression analysis for the nattokinase activities according to the Box-Behnken experimental design

| Source | SS | DF | MS | F Value | *p* | Significance |
| --- | --- | --- | --- | --- | --- | --- |
| Model | 6.297E+0.05 | 14 | 44975.89 | 47.52 | <0.0001 | *** |
| *A* | 76399.50 | 1 | 76399.50 | 80.72 | <0.0001 | *** |
| *B* | 1.080E+0.05 | 1 | 1.080E+0.05 | 114.14 | <0.0001 | *** |
| *C* | 9256.01 | 1 | 9256.01 | 9.78 | 0.0074 | ** |
| *D* | 76282.43 | 1 | 76282.43 | 80.60 | <0.0001 | *** |
| *AB* | 3080.25 | 1 | 3080.25 | 3.25 | 0.0928 |  |
| *AC* | 132.25 | 1 | 132.25 | 0.14 | 0.7142 |  |
| *AD* | 961.00 | 1 | 961.00 | 1.02 | 0.3307 |  |
| *BC* | 5550.25 | 1 | 5550.25 | 5.86 | 0.0296 |  |
| *BD* | 600.25 | 1 | 600.25 | 0.63 | 0.4391 |  |
| *CD* | 1089.00 | 1 | 1089.00 | 1.15 | 0.3016 |  |
| *A^2^* | 3.600E+0.05 | 1 | 3.600E+0.05 | 380.35 | <0.0001 | *** |
| *B^2^* | 1.644E+0.05 | 1 | 1.644E+0.05 | 173.71 | <0.0001 | *** |
| *C^2^* | 1.535E+0.05 | 1 | 1.535E+0.05 | 162.18 | <0.0001 | *** |
| *D^2^* | 1.991E+0.05 | 1 | 1.991E+0.05 | 210.38 | <0.0001 | *** |
| Residual | 13250.75 | 14 | 946.48 |  |  |  |
| Lack of fit | 10084.75 | 10 | 1008.47 | 1.27 | 0.4396 |  |
| Pure error | 3166.00 | 4 | 791.50 |  |  |  |
| Cor total | 6.429E+0.05 | 28 |  |  |  |  |

Note：^*^ *p* < 0.05；^**^ *p* < 0.01；^***^ *p* < 0.001

*R^2^* = 0.9794，Adj *R^2^* = 0.9588

SS: Sum of squares; DF: Degree freedom; MS: Mean square.
